# Reduced TRMU expression increases the sensitivity of hair-cell-like HEI-OC-1 cells to neomycin damage in vitro

**DOI:** 10.1038/srep29621

**Published:** 2016-07-13

**Authors:** Zuhong He, Shan Sun, Muhammad Waqas, Xiaoli Zhang, Fuping Qian, Cheng Cheng, Mingshu Zhang, Shasha Zhang, Yongming Wang, Mingliang Tang, Huawei Li, Renjie Chai

**Affiliations:** 1 State Key Laboratory of Bioelectronics, Institute of Life Sciences, Southeast University, Nanjing 210096, China; 2 Department of Otorhinolaryngology, Hearing Research Institute, Affiliated Eye and ENT Hospital of Fudan University, Shanghai 200031, China; 3 MOE Key Laboratory of Developmental Genes and Human Disease, Institute of Life Sciences, Southeast University, Nanjing 210096, China; 4 Co-Innovation Center of Neuroregeneration, Nantong University, Nantong 226001, China; 5 Department of Otolaryngology, Affiliated Drum Tower Hospital of Nanjing University Medical School, Nanjing 210008, China; 6 Medical School, Southeast University, Nanjing 210096, China; 7 Institutes of Life Sciences, Fudan University, Shanghai 200032, China

## Abstract

Aminoglycosides are ototoxic to the cochlear hair cells, and mitochondrial dysfunction is one of the major mechanisms behind ototoxic drug-induced hair cell death. TRMU (tRNA 5-methylaminomethyl-2-thiouridylate methyltransferase) is a mitochondrial protein that participates in mitochondrial tRNA modifications, but the role of TRMU in aminoglycoside-induced ototoxicity remains to be elucidated. In this study, we took advantage of the HEI-OC-1 cell line to investigate the role of TRMU in aminoglycoside-induced cell death. We found that TRMU is expressed in both hair cells and HEI-OC-1 cells, and its expression is significantly decreased after 24 h neomycin treatment. We then downregulated TRMU expression with siRNA and found that cell death and apoptosis were significantly increased after neomycin injury. Furthermore, when we down-regulated TRMU expression, we observed significantly increased mitochondrial dysfunction and increased levels of reactive oxygen species (ROS) after neomycin injury, suggesting that TRMU regulates mitochondrial function and ROS levels. Lastly, the antioxidant N-acetylcysteine rescued the mitochondrial dysfunction and cell apoptosis that was induced by TRMU downregulation, suggesting that ROS accumulation contributed to the increased aminoglycosides sensitivity of HEI-OC-1 cells after TRMU downregulation. This study provides evidence that TRMU might be a new therapeutic target for the prevention of aminoglycoside-induced hair cell death.

Aminoglycoside antibiotics are widely used throughout the world, but while they are highly effective against gram-negative bacterial infections, aminoglycoside-induced hair cell damage is one of the most common causes of hair cell death^1^. Thus, despite their usefulness, these drugs are frequently ototoxic^2^ and induce apoptosis in hair cells through oxidative stress^3^. The genes regulating the ototoxic sensitivity of hair cells are largely unknown, and the mechanisms involved in ototoxic sensitivity are not well understood.

Mitochondria are cellular organelles that regulate major cellular processes, including cellular metabolism, communication, development, and apoptosis. Recently, mutations in mitochondrial DNA (mtDNA) have been reported to be one cause of sensorineural hearing loss^4^. These mutations in the mtDNA and abnormal translation of mitochondrial genes induce destructive cellular mechanisms, including mitochondrial dysfunction^5^, increased oxidative stress^4^, reduced mitochondrial translation^6^, diminished activity of respiratory enzymes, and decreased oxygen consumption^7^,^8^. Abnormal mitochondrial translation is frequently caused by mutations in nuclear genes encoding tRNA modifying factors and mt-tRNA aminoacyl-synthetase^9^. Other nuclear genes that are implicated in mitochondrial diseases in various organs include the nuclear-encoded mitochondrial transcription factor B1 (*TFB1M*)^10^, GTP-binding protein 3 (*GTPBP3*)^11^, mitochondrial translational optimization protein 1 (*MTO1*)^12^, and the mitochondrial tRNA-specific modification enzyme tRNA 5-methylaminomethyl-2-thiouridylate methyltransferase (*TRMU*)^13^.

The *TRMU* gene (also known as *MTU1* or *MTO2*) encodes a protein that is a putative nuclear modifier of deafness-associated mtDNA mutations^13^,^14^. The TRMU protein optimizes the translation process by modifying the 2-thio positions of the wobble U in tRNA^15^,^16^. This modification of adding a 2-thio group to the wobble base of the uridine at position 34 of tRNA is crucial for accurate and efficient synthesis of several mtDNA-encoded proteins involved in the respiratory chain. The loss of post-transcriptional modification in the wobble positions of mt-tRNAs causes stroke-like episodes (MELAS), mitochondrial encephalomyopathy, myoclonic epilepsy with ragged-red fibers (MERRF), and lactic acidosis^17^. Prior studies in yeast have demonstrated that mutations in TRMU impair the steady-state levels of mitochondrial tRNAs^18^ and modify the process of adding 2-thio groups to Lys, Glu, and Gln at the mt-tRNA wobble base position. Recent studies have reported that a lack of the 2-thio modification by TRMU is associated with acute infantile liver failure^16^,^19^, which indicates that TRMU might have some relation with apoptosis.

The molecular mechanism behind the apoptosis induced by neomycin (an aminoglycoside antibiotic) has been well studied^20^,^21^,^22^,^23^,^24^; however, the role of TRMU in neomycin-induced mitochondrial dysfunction and hair cell apoptosis is still not fully known. The production of reactive oxygen species (ROS) from mitochondria has been reported to be a major factor in aminoglycoside-induced hair cell damage by stimulating apoptosis in the cochlea^25^,^26^. Nevertheless, the role of mt-tRNA modifying enzymes in neomycin-induced hair cell apoptosis and concurrent ROS accumulation needs to be further investigated. In this study, we have used the hair-cell-like HEI-OC-1 cell line to investigate the role of TRMU in aminoglycoside-induced cell death. The HEI-OC-1 cell line has been used as a model system for the cochlea in numerous studies, and these cells express several molecular markers of cochlear hair cells, including calbindin, calmodulin, Math 1, Myosin 7a, and prestin^27^,^28^.

In the present study, we demonstrate the role of TRMU in neomycin-induced apoptosis in the HEI-OC-1 cell line. We show that downregulation of TRMU by siRNA in neomycin-treated HEI-OC-1 cells promotes apoptosis through mitochondrial dysfunction and ROS accumulation. Our study highlights the role of TRMU in regulating the sensitivity of HEI-OC-1 cells to neomycin-induced damage.

## Results

### TRMU is expressed in the cochlear hair cells and HEI-OC-1 cells, and the expression of TRMU is reduced after neomycin damage

**Figure 1:**
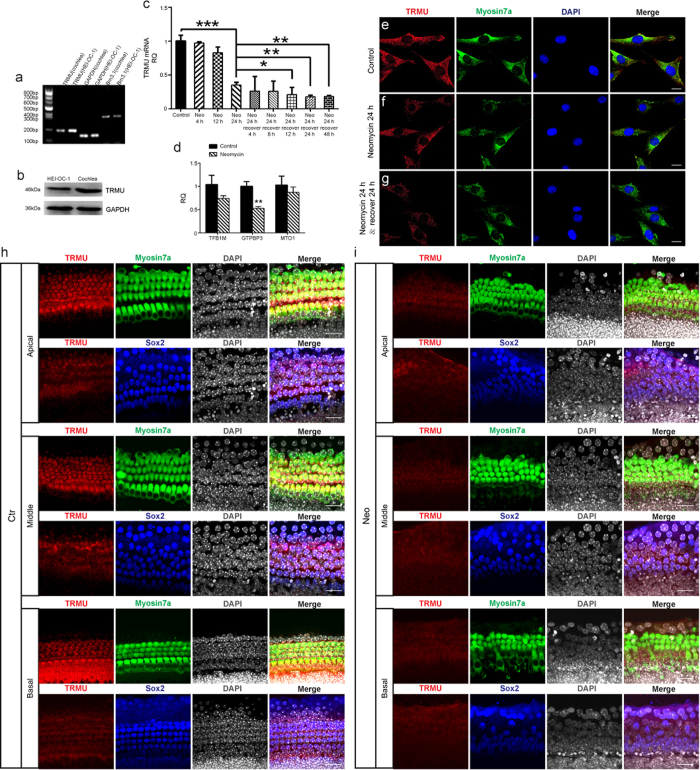
Decreased TRMU expression in HEI-OC-1 cells after neomycin treatment at different time points. (**a**) RT-PCR results showed that TRMU is expressed in the mouse cochlea and HEI-OC-1 cells. (**b**) Western blot results showed that TRMU is expressed in the mouse cochlea. (**c**) The cells were treated with neomycin for 4 h, 12 h, or 24 h, allowed to recover for 0 h, 4 h, 8 h, 12 h, 24 h, or 48 h, and collected for analysis of TRMU expression by qPCR. (**d**) The mRNA levels of other mitochondrial genes in the control group and the neomycin-treatment group. (**e**–**g**) Immunofluorescence staining in the cochlea with TRMU and myosin-7a antibodies. The results showed that the expression of TRMU was significantly decreased with neomycin treatment followed by 24 h recovery. (**h**,**i**) Consistent with the results in the HEI-OC-1 cell line, immunofluorescence staining in the cochlea showed immunoreactivity against TRMU, myosin-7a, and Sox2. The expression of TRMU in hair cells was significantly decreased after neomycin treatment followed by 24 h recovery. For all experiments, the values for normal controls were set to 1. Scale bars = 20 μm, *p < 0.05, **p < 0.01, ***p < 0.001.

Previous reports show that TRMU is expressed in tissues with high levels of oxidative metabolism^29^,^30^. Here we first investigated the expression of TRMU in the mouse cochlea. RT-PCR and western blot data demonstrated that TRMU is highly expressed in the cochlea (Fig. 1a,b). Immunohistochemistry data further showed that TRMU is expressed in both hair cells and supporting cells in all three turns of the cochlea, and the expression of TRMU in supporting cells is much lower than in hair cells (Fig. 1h). RT-PCR, western blot, and immunohistochemistry results confirmed that TRMU is expressed in the HEI-OC-1 cells (Fig. 1a,b,e). To determine the optimum neomycin treatment for inducing hair cell damage, we tested different neomycin concentrations (0.2 mM to 100 mM), treatment times (4 h to 48 h), and recovery times (4 h to 48 h) (Supplemental Figure 1a–j). We found that 10 mM neomycin treatment for 24 h led to an appropriate level of damage to the HEI-OC-1 cells (there were insufficient numbers of cells remaining for the following experiments when 10 mM neomycin was added for 48 h), and we used this neomycin concentration for all the experiments in this study.

To further examine the expression of TRMU in HEI-OC-1 cells after neomycin treatment, we exposed the HEI-OC-1 cells to 10 mM neomycin for three different times (4 h, 12 h, and 24 h). qPCR results showed that the 24 h neomycin treatment significantly decreased the expression of TRMU (Fig. 1c; *p* < 0.001). After 24 h neomycin treatment, we washed away the neomycin and let the HEI-OC-1 cells recover for 4 h, 8 h, 12 h, 24 h, or 48 h. qPCR results showed that after 24 h recovery, the expression of TRMU was further reduced (Fig. 1c; *p < 0.05, **p < 0.01). Immunofluorescence staining with anti-TRMU and anti-Myosin7a antibodies confirmed that after 24 h neomycin treatment the TRMU expression was significantly reduced (Fig. 1f), and after 24 h recovery the TRMU expression was further down-regulated (Fig. 1g). Immunoflourescence staining experiments were also performed to validate the expression of TRMU after neomycin treatment. We found that the expression of TRMU in hair cells was significantly reduced when treated with neomycin for 24 h followed by 24 h recovery (Fig. 1h,i), while the expression of TRMU in supporting cells was not significantly changed after neomycin treatment (Fig. 1h,i). In addition, we performed a qPCR experiment to validate the expression of other mitochondrial genes, including *TFB1M*, *GTPBP3*, and *MTO1*, after 24 h neomycin treatment followed by 24 h recovery. The results showed that the expression levels of these three genes were all decreased after neomycin treatment (Fig. 1d). However, only the decreased expression of *GTPBP3* was statistically significant when compared with the control cells. Together, these results suggest that neomycin injury significantly downregulates the expression of TRMU in cochlear hair cells and HEI-OC-1 cells.

### siRNA downregulates the expression of TRMU in HEI-OC-1 cells

**Figure 2:**
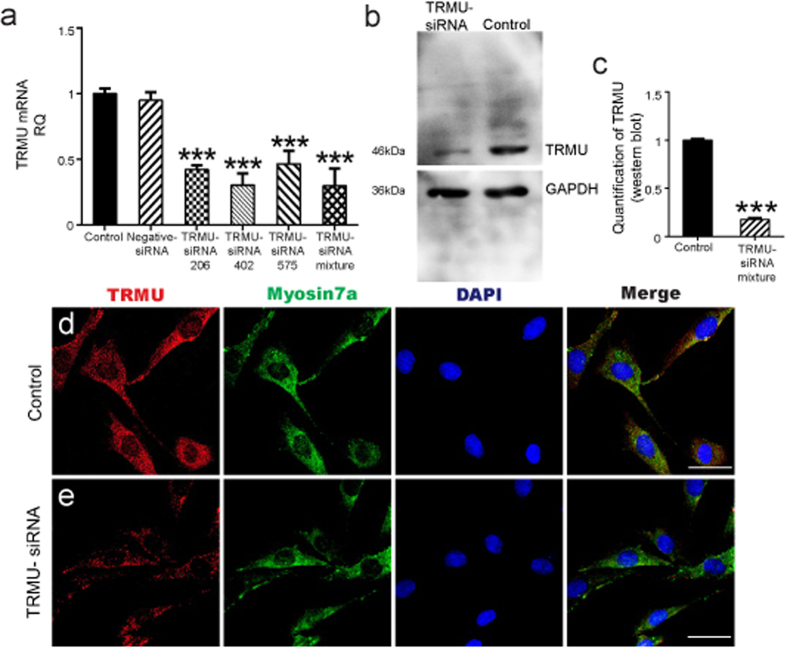
Downregulation of TRMU by transfection with TRMU-siRNA. (**a**) The mRNA levels of TRMU decreased significantly after transfection with each TRMU-siRNA and the mixture of all three TRMU-siRNAs. (**b**) The protein levels of TRMU decreased significantly after transfection with the TRMU-siRNA mixture. (**c**) Semi-quantification of the western blot in b. (**d**,**e**) Immunofluorescence staining showing that the expression of TRMU was significantly decreased after transfecting with the TRMU-siRNA mixture. For all experiments, the values for normal controls were set to 1. Scale bars = 20 μm, *p < 0.05, **p < 0.01, ***p < 0.001.

Exposure to neomycin induced high levels of caspase 3 activation in the HEI-OC1 cell line, while the function of TRMU is to maintain the high fidelity of codon recognition and the formation and stabilization of functional tRNA structures. Thus, TRMU might be involved in the neomycin-induced damage in HEI-OC1 cells. In order to investigate the role of TRMU in neomycin-induced cell death in the HEI-OC-1 cell line, we knocked down TRMU by siRNA. First, we measured the efficiency of the transfection system using nonsense siRNA conjugated with 6′-carboxyfluorescein (FAM). We found that 93.4% of all DAPI-positive cells were also FAM positive, suggesting that 93.4% of the HEI-OC-1 cells were successfully transfected with FAM-siRNA (Supplemental Figure 2). We designed three TRMU-siRNA constructs (siRNA-206, siRNA-402, siRNA-575) and used them to transfect the HEI-OC-1 cell line. qPCR results showed that TRMU expression was significantly reduced after transfection with siRNA-206, siRNA-402, siRNA-575, and all three siRNAs combined. The lowest TRMU expression was observed when HEI-OC-1 cells were transfected with the mixture of all three siRNAs (Fig. 2a; *p* < 0.001), thus we used the siRNA mixture in all of the following experiments. To further confirm our findings, we performed western blot experiments and found significantly decreased expression of TRMU protein after transfection with the siRNA mixture (Fig. 2b). The semi-quantification of western blot data suggested that the expression of TRMU was decreased to 21 ± 1.37% that of the controls after transfection with the siRNA mixture (Fig. 2c; *p* < 0.001). Moreover, the immunofluorescence staining also showed that the expression of TRMU in HEI-OC-1 cells was significantly downregulated after transfection with the siRNA mixture when compared with the control group (Fig. 2d,e).

### Downregulation of TRMU increases cell death and apoptosis in HEI-OC-1 cells after neomycin exposure

**Figure 3:**
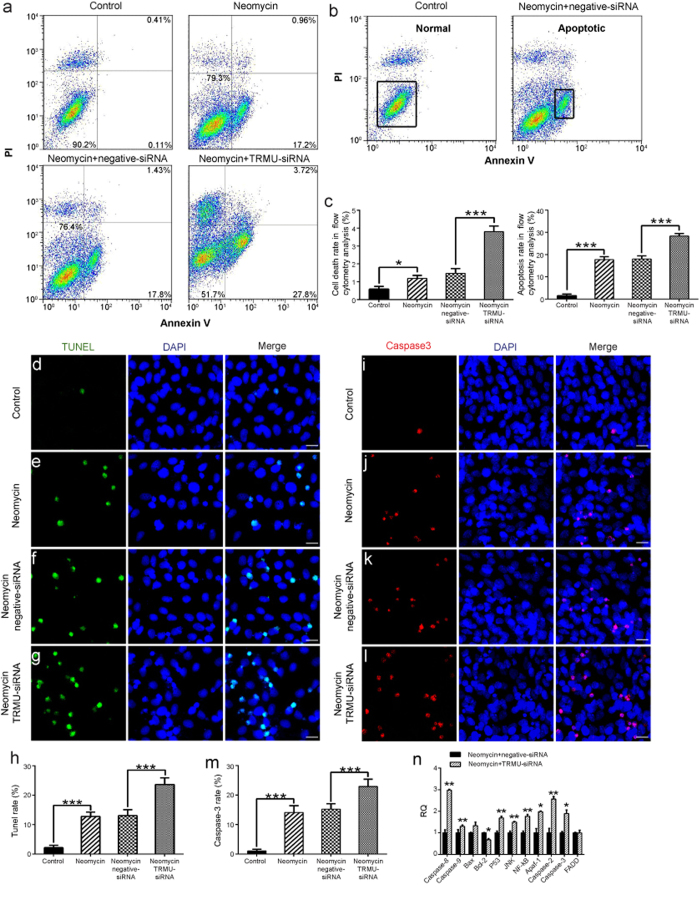
Downregulation of TRMU expression in HEI-OC-1 cells increased their sensitivity to neomycin damage. (**a**) Apoptosis analysis by flow cytometry. The lower left quadrants, lower right quadrants, and upper right quadrants of the images represent live cells, early apoptotic cells, and late apoptotic cells, respectively. (**b**) The left panel represents live cells in the control group without neomycin damage, and the right panel represents neomycin-induced early apoptotic cells in the negative-siRNA transfection group. (**c**) The flow cytometry results showed that with neomycin damage, the proportions of dead cells and early apoptotic cells were significantly greater after TRMU-siRNA knock down compared with the negative-siRNA transfection group. (**d**–**g**) TUNEL and DAPI double staining showing the apoptotic HEI-OC-1 cells. (**i**–**l**) Cleaved caspase-3 and DAPI double staining showing the increased apoptotic cells after TRMU downregulation and neomycin treatment. (**h,i**) The number of TUNEL^+^/DAPI^+^ and cleaved caspase-3^+^/DAPI^+^ cells increased significantly after downregulation of TRMU. (**n**) With neomycin treatment, the mRNA levels of eleven apoptosis-related genes were analyzed by qPCR, and among them the expression levels of eight pro-apoptotic genes were significantly higher while one of the anti-apoptotic genes (*BCL2*) showed decreased expression. For the qPCR experiments, the values for the normal controls were set to 1. Scale bars = 20 μm, *p < 0.05, **p < 0.01, ***p < 0.001.

First we investigated whether downregulation of TRMU alone leads to cell apoptosis and mitochondrial damage without neomycin treatment. We quantified the cell apoptosis in electron micrographs by assessing the common apoptotic features in cells such as smaller cell bodies, cytoplasmic concentration, nuclear chromatin condensation, increased apoptotic bodies, and membrane vesicle formation. Our data showed that knockdown of TRMU expression alone does not cause significant apoptosis or mitochondrial structural damage in HEI-OC-1 cells without neomycin damage (Supplemental Figure 3a–c). Next, we knocked down TRMU expression with siRNA to investigate the role of TRMU in the neomycin-induced cell death of HEI-OC-1 cells. In this experiment, the control HEI-OC-1 cells and the TRMU-siRNA–transfected HEI-OC-1 cells were treated with 10 mM neomycin. After 24 h of neomycin treatment, the cells were washed with phosphate buffered solution (PBS) and allowed to recover in culture medium for another 24 h. We used propidium iodide (PI) to label the dead cells and Annexin V to label the cells undergoing apoptosis (Fig. 3a,b). We found that the numbers of both dead and apoptotic cells were significantly increased after neomycin treatment compared to the undamaged control cells (Fig. 3a,c; *p < 0.05, ***p < 0.001). Notably, after neomycin injury the cells transfected with the siRNA mixture had a significantly greater ratio of dead cells to apoptotic cells than the controls transfected with negative siRNA (Fig. 3a,c; *p* < 0.001).

Previous studies have reported that cleaved caspase-3 and TUNEL can be used as markers of apoptosis in aminoglycoside-induced hair cell death^31^,^32^,^33^. We performed caspase-3 and TUNEL staining to detect the apoptotic HEI-OC-1 cells after 24 h neomycin treatment followed by 24 h recovery. We found significantly more caspase-3-positive and TUNEL-positive cells in the neomycin-treated groups compared with the undamaged controls (Fig. 3d,e,i and j; *p* < 0.001). In particular, the siRNA-transfected cells had significantly more caspase-3-positive and TUNEL-positive cells compared to the controls transfected with negative siRNA (Fig. 3f–h and 3k–m; *p* < 0.001). qPCR results showed that with neomycin treatment the expression of pro-apoptotic marker genes, including *CASP8*, *CASP9*, *p53*, *JNK*, *NF-kB*, *Apaf-1*, *CASP2*, and *CASP3*, were significantly higher in TRMU-siRNA–transfected cells than controls transfected with negative siRNA (Fig. 3n; *p < 0.05, **p < 0.01). Concurrently, the expression of *BCL2*, which is an anti-apoptotic gene, was significantly lower in TRMU-siRNA–transfected cells (Fig. 3n, *p* < 0.05). Without neomycin treatment, the expression of pro-apoptotic factors in the TRMU-siRNA–transfected groups showed no differences compared to the undamaged control groups (Supplemental Figure 3c). Taken together, these results demonstrated that knockdown of TRMU by siRNA significantly increased the cell death and apoptosis of HEI-OC-1 cells after neomycin injury, indicating that downregulation of TRMU increases the sensitivity of HEI-OC-1 cells to neomycin damage.

### Downregulation of TRMU reduces the mitochondrial membrane potential and affects mitochondrial function after neomycin injury

**Figure 4:**
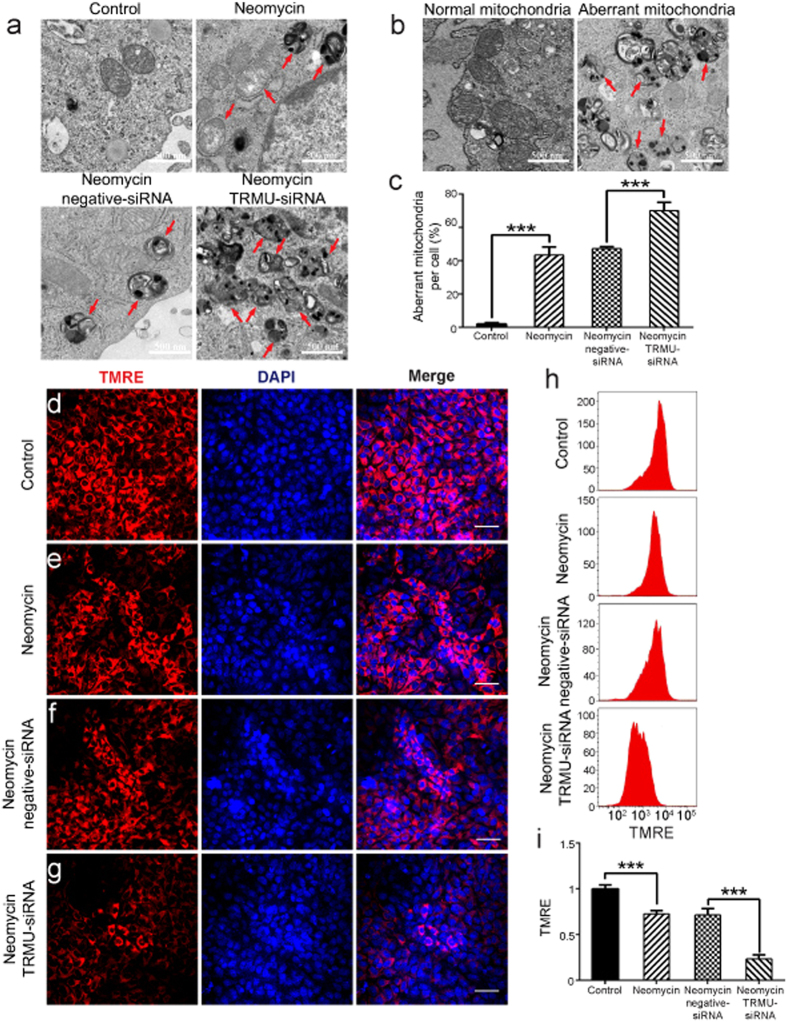
TRMU downregulation affects the mitochondrial membrane potential after neomycin treatment. (**a**) Transmission Electron Microscope analysis (TEM) to evaluate mitochondrial function. The results showed that the number of aberrant mitochondria was significantly increased after TRMU downregulation along with neomycin treatment for 24 h. (**b**) Mitochondrial structural damage (such as vacuolated mitochondria, the appearance of myelin bodies, and mitochondrial cristae that had decrease in number, become discontinuous, or disappeared completely) is indicated by black arrows in HEI-OC-1 cells. (**c**) Quantification of the results in a. (**d**–**g**) Four different groups of HEI-OC-1 cells were labeled using the TMRE staining kit. The results showed that the mitochondrial membrane potential was significantly decreased after TRMU downregulation along with neomycin treatment for 24 h. (**h**) The analysis of mitochondrial membrane potential by flow cytometry. (**i**) Quantification of the data in h. For all experiments, the values for the normal controls were set to 1. Scale bars = 20 μm, *p < 0.05, **p < 0.01, ***p < 0.001.

To further explore the detailed mechanism behind the increased sensitivity of HEI-OC-1 cells to neomycin treatment after TRMU knockdown, we took advantage of transmission electron microscope analysis (TEM) to evaluate mitochondrial function and used the TMRE kit to quantify changes in the mitochondrial membrane potential. In this experiment, the control HEI-OC-1 cells and the TRMU-siRNA–transfected cells were treated with 10 mM neomycin. After 24 h of neomycin treatment and 24 h recovery, the cells were collected to perform TEM or immunofluorescence staining. TEM data showed that without neomycin treatment the TRMU-siRNA–transfected groups showed no difference compared to the undamaged control groups (Supplemental Figure 3a–c). In addition, we found aberrant mitochondrial structures, such as vacuolated mitochondria, the appearance of myelin bodies, and mitochondrial cristae that had decreased in number, become discontinuous, or completely disappeared (Fig. 4b). Non-transfected but neomycin-treated groups had significantly more mitochondrial structural abnormalities compared with the undamaged controls (Fig. 4a,b,c; *p* < 0.001), and the TRMU-siRNA–transfected groups had significantly more aberrant mitochondria compared to the negative siRNA controls (Fig. 4a,c; *p* < 0.001). Immunohistochemistry and flow cytometry data showed that TMRE intensity was significantly decreased after neomycin treatment compared with the undamaged controls (Fig. 4d–i; *p* < 0.001), and TMRE intensity was significantly decreased in the TRMU-siRNA–transfected groups compared with the negative siRNA controls (Fig. 4d–i; *p* < 0.001). Together, these findings suggested that TRMU downregulation exacerbated the mitochondrial dysfunction in HEI-OC-1 cells after neomycin injury.

### Downregulation of TRMU increases intracellular ROS levels after neomycin injury

**Figure 5:**
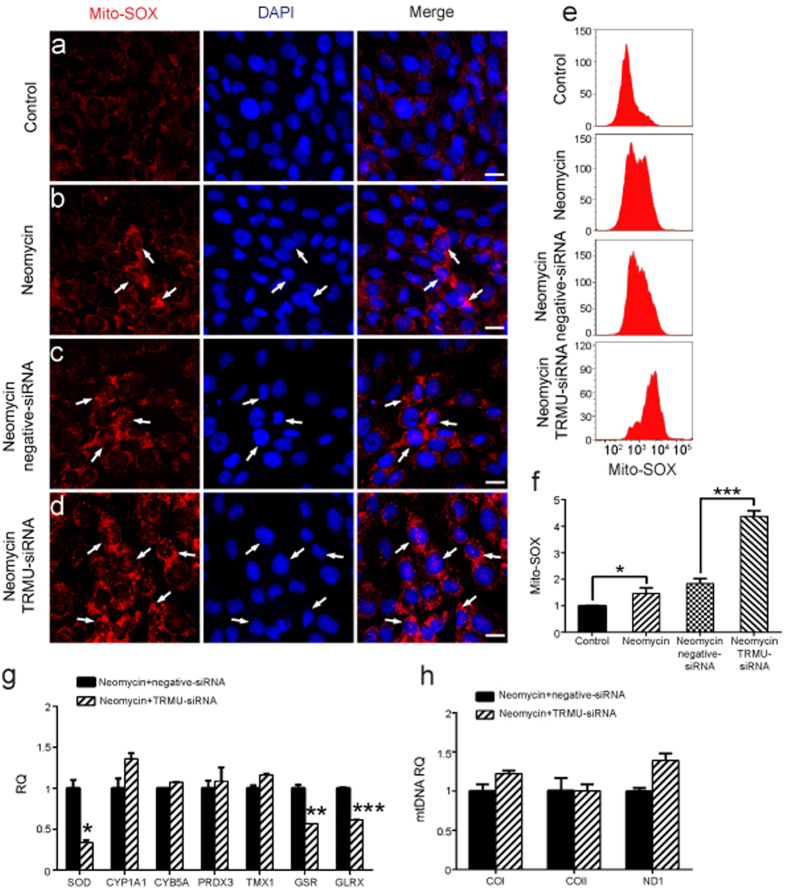
The changes in ROS levels in HEI-OC-1 cells after transfection with TRMU-siRNA followed by neomycin treatment. (**a**–**d**) Four different groups of HEI-OC-1 cells were labeled using the Mito-SOX staining kit. The results showed that the ROS levels were significantly increased after transfection with TRMU-siRNA followed by neomycin treatment for 24 h and 24 h recovery. (**e**) Flow cytometry data confirmed the results in (**a**–**d)**. (**f**) Quantification of the data in e. (**g**) The mRNA levels of seven genes related to oxidation-reduction reactions were analyzed by qPCR, and the expression of three genes (*SOD*, *GSR*, and *GLRX*) was significantly decreased. (**h**) The mtDNA copy number was analyzed by qPCR using the *ND1*, *COI*, and *COII* genes as representative of mtDNA copy number. No changes were observed in HEI-OC-1 cells after siRNA transfection and neomycin treatment. For all experiments, the values for the normal controls were set to 1. Scale bars = 20 μm, *p < 0.05, **p < 0.01, ***p < 0.001.

ROS have been reported to play important roles in aminoglycoside-induced hair cell damage^34^,^35^. To evaluate mitochondrial ROS levels in neomycin-treated HEI-OC-1 cells after downregulation of TRMU, we used Mito-SOX Red, which is a redox fluorophore that selectively detects mitochondrial superoxide^36^,^37^. Here the control HEI-OC-1 cells and the TRMU-siRNA–transfected cells were treated with 10 mM neomycin for 24 h and allowed to recover for 24 h. Cells were then collected for immunohistochemistry and flow cytometry. Immunohistochemistry and flow cytometry results showed that the ROS levels were increased after neomycin treatment compared to the undamaged controls (Fig. 5a,b,e,f; *p* < 0.05), and downregulation of TRMU by siRNA transfection significantly increased the ROS levels in HEI-OC-1 cells compared with the negative siRNA controls (Fig. 5c,d,e,f; *p* < 0.001).

To further verify our findings, we analyzed the mRNA expression of seven redox-related genes by qPCR and found significantly decreased expression of three important antioxidant genes, including *SOD*, *GSR*, and *GLRX*, in HEI-OC-1 cells after TRMU downregulation followed by neomycin treatment (Fig. 5g; *p < 0.05, **p < 0.01, ***p < 0.001). Prior studies showed that an elevated ROS level induces the cell apoptosis pathway by downregulating the mtDNA copy number^37^,^38^. To detect the mtDNA copy number after TRMU downregulation, we measured the expression of three mtDNA marker genes – *ND1*, *COI*, and *COII*. However, the qPCR data showed no significant difference in mtDNA copy number in HEI-OC-1 cells after TRMU downregulation as compared with the control cells (Fig. 5h). Together, these results demonstrated that TRMU downregulation inhibited the expression of antioxidant genes and increased the intracellular ROS levels in HEI-OC-1 cells after neomycin injury.

### Antioxidant treatment rescues the cell death and apoptosis induced by TRMU downregulation after neomycin injury

To investigate whether the elevated ROS level contributes to the increased sensitivity of HEI-OC-1 cells to neomycin damage after TRMU downregulation, we treated the HEI-OC-1 cells with the antioxidant N-acetylcysteine (NAC), which is a reduced glutathione provider and a direct scavenger of reactive oxygen intermediates. First, we determined the optimum concentration of NAC in HEI-OC-1 cells. In this experiment, after TRMU-siRNA transfection for 24 h, the cells were pretreated with different concentrations of NAC (from 1 mM to 100 mM) for 12 h or 24 h. The HEI-OC-1 cells were then treated with 10 mM neomycin for 24 h and allowed to recover for 24 h together with NAC. Cell numbers were determined with the CCK-8 kit. The results showed that the cell number reached its peak with the 2 mM NAC treatment for 24 h (Supplemental Figure 4a,b,c; *p* < 0.01). To confirm these results, we performed a flow cytometry experiment and found that the numbers of both dead and apoptotic cells in the pre-treated 2 mM NAC 24 h group were significantly reduced after neomycin treatment compared to the cells exposed to neomycin without NAC pre-treatment. Thus pre-treatment with 2 mM NAC for 24 h was used for all subsequent experiments (Supplemental Figure 4d,e; **p < 0.01, ***p < 0.001). Mito-SOX red was used to measure the changes in ROS levels in the HEI-OC-1 cells after NAC treatment. The immunohistochemistry and flow cytometry results showed that the ROS level was significantly decreased after neomycin treatment when the TRMU-siRNA–transfected HEI-OC-1 cells were pretreated with 2 mM NAC for 24 h (Supplemental Figure 5; *p* < 0.001), suggesting that NAC treatment could efficiently reduce the ROS level in HEI-OC-1 cells.

**Figure 6:**
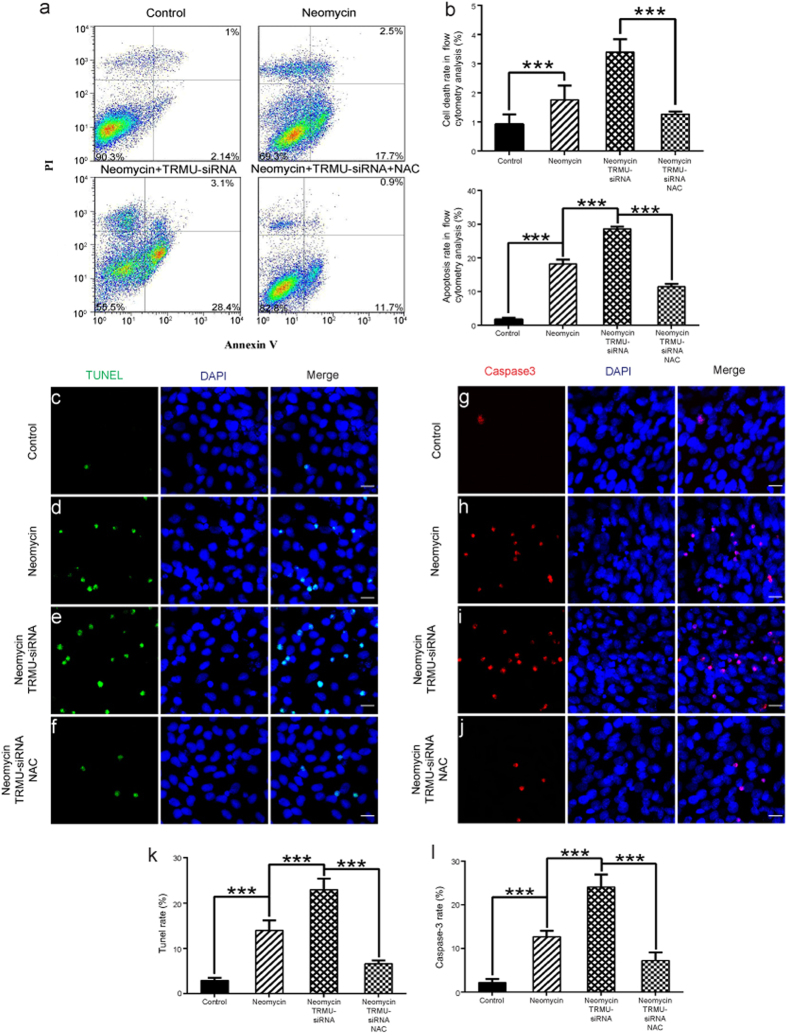
Influence of antioxidants on the apoptosis of TRMU-downregulated HEI-OC-1 cells after neomycin treatment. (**a**) Analysis of apoptotic HEI-OC-1 cells by flow cytometry after pretreatment with NAC. The proportion of apoptotic cells after TRMU downregulation significantly decreased when the increased ROS level was blocked by NAC, although the cell count was slightly increased compared with the control cells. (**b**) The numbers of dead cells and early apoptotic cells were significantly lower after pretreatment with NAC. (**c**–**f**) TUNEL and DAPI double staining and (**g**–**j**) cleaved caspase-3 and DAPI double staining showed the number of apoptotic HEI-OC-1 cells. (**k**,**l**) The numbers of TUNEL^+^/DAPI^+^ and cleaved caspase-3^+^/DAPI^+^ cells significantly decreased after pretreatment with NAC. For qPCR experiments, the values for the normal controls were set to 1. Scale bars = 20 μm, *p < 0.05, **p < 0.01, ***p < 0.001.

We next investigated the changes in the rates of cell death and apoptosis of HEI-OC-1 cells when pretreated with NAC. Our results showed that when pretreated with NAC, the cell death and apoptosis rates were significantly decreased in TRMU-siRNA–transfected HEI-OC-1 cells (Fig. 6a,b; *p* < 0.001). Immunostaining for caspase-3 and TUNEL was performed to confirm our findings, and we observed significantly fewer caspase-3/DAPI and TUNEL/DAPI double-positive cells after NAC treatment (Fig. 6c–l; *p* < 0.001). These results demonstrated that ROS accumulation plays an important role in the increased sensitivity of HEI-OC-1 cells to neomycin damage after TRMU downregulation.

### Antioxidant treatment rescued the dysfunction of mitochondria induced by TRMU downregulation after neomycin injury

**Figure 7:**
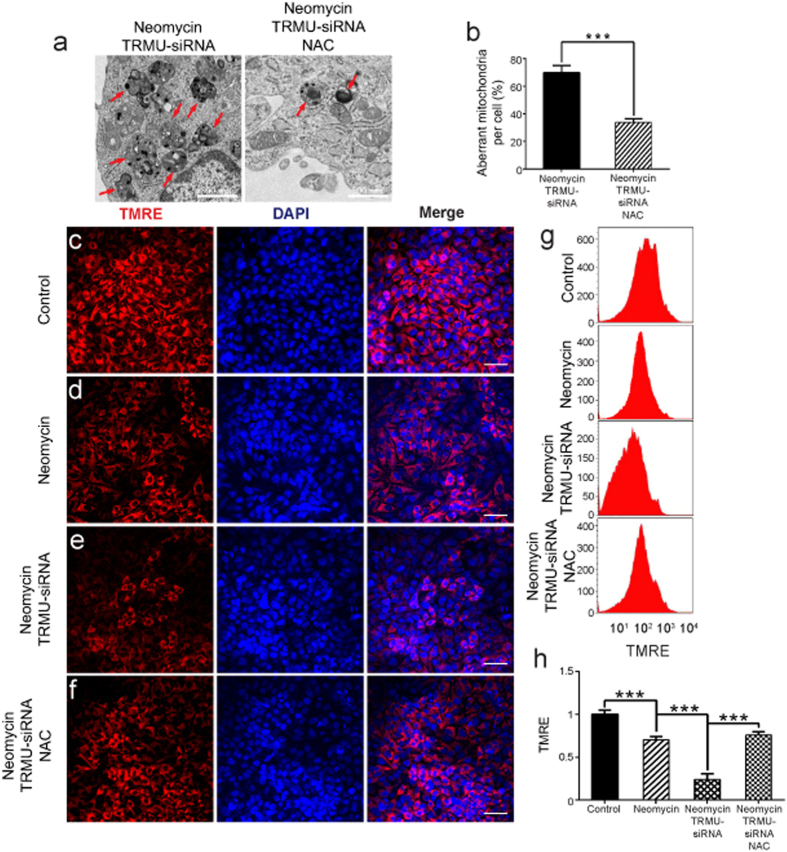
The influence of antioxidants on the mitochondrial membrane potential of TRMU-downregulated HEI-OC-1 cells after neomycin treatment. (**a**) Transmission Electron Microscope analysis (TEM) to evaluate mitochondrial function. The results showed that the numbers of aberrant mitochondria were significantly decreased when the increase in ROS level was blocked by NAC, although there was still a small number of residual aberrant mitochondria. (**b**) Quantification of the TEM data. (**c**–**f**) Four different groups of HEI-OC-1 cells were labeled with the TMRE kit. The results showed that the mitochondrial membrane potential was significantly increased after pretreatment with NAC, but the level was slightly decreased compared with the control cells. (**g**) Flow cytometry data confirmed the results in (**c**–**f**). (**h**) Quantification of the flow cytometry data. For all experiments, the values for the normal controls were set to 1. Scale bars = 20 μm, *p < 0.05, **p < 0.01, ***p < 0.001.

To further explore whether the elevated ROS level contributes to the increased mitochondrial dysfunction of HEI-OC-1 cells after TRMU downregulation, we used TEM and TMRE staining to investigate the effects of NAC treatment on the mitochondrial function of HEI-OC-1 cells after TRMU downregulation. When pretreated with 2 mM NAC, the TEM results showed that the numbers of aberrant mitochondria were significantly decreased in TRMU-siRNA–transfected HEI-OC-1 cells (Fig. 7a,b; *p* < 0.001), and the immunohistochemistry and flow cytometry results showed that the TMRE intensity was significantly increased in TRMU-siRNA–transfected HEI-OC-1 cells (Fig. 7c–h; *p* < 0.001). These results suggested that NAC treatment significantly reduced the mitochondrial dysfunction caused by TRMU downregulation after neomycin injury.

## Discussion

All aminoglycosides have the side effects of nephrotoxicity, ototoxicity, and neuromuscular blockade, and these limit the clinical use of aminoglycoside antibiotics^2^,^39^. Aminoglycoside ototoxicity is typically associated with bilateral sensorineural hearing loss because the sensory hair cells are susceptible to aminoglycoside-induced cytotoxicity and are not able to regenerate once they are damaged^40^.

The nuclear-modifier gene *TRMU* has been reported to modulate the phenotypic manifestation of mitochondrial defects in multiple organs^41^,^42^, and recent research has shown that mutations in *TRMU* increase the risk of deafness and transient infantile liver failure^41^. Loss of *TRMU* function has been shown to cause defective thiolation of the third anticodon positions on mitochondrial tRNA Lys, tRNA Glu, and tRNA Gln, and these aggravate the respiratory deficiency of the C1409G mutation that is associated with human deafness^13^,^43^. Guan *et al*. determined that *TRMU* is a putative nuclear modifier gene that can modulate the phenotypic expression of deafness-associated mitochondrial 12S rRNA mutations^13^. The mutational analysis performed in Arab-Israeli and European families identified a single missense mutation in *TRMU* leading to an A10S substitution in the TRMU protein. The frequency of the TRMU A10S variant was 25% in Arab-Israeli and European families, who also carried the 12S rRNA A1555G mutation. The persons carrying both the homozygous TRMU A10S and A1555G mutations exhibited prelingual profound deafness, while the TRMU A10S mutation alone, even in a homozygous form, was not sufficient to cause a hearing loss^13^,^30^. In this study, and consistent with previous reports, we also found that down-regulation of TRMU does not decrease HEI-OC1 cell viability without neomycin treatment (Supplemental Figure 3). Next, we explored the role of TRMU in neomycin-induced damage in hair-cell-like HEI-OC-1 cells. We first reported the expression of TRMU in the hair cells and HEI-OC-1 cells, which is downregulated after neomycin injury (Fig. 1). We then showed that downregulation of TRMU dramatically increased the cell death and apoptosis of HEI-OC-1 cells after neomycin injury (Fig. 3). In addition, when we downregulated the TRMU expression, we observed increased expression of seven pro-apoptotic genes, including *CASP8*, *CASP9*, *p53*, *JNK*, *NF-kB*, *Apaf-1*, *CASP2*, *CASP3*, and decreased expression of the anti-apoptotic gene *BCL2* after neomycin injury, which suggests a role for TRMU in the cell apoptotic pathway. Lastly, we demonstrated that treatment with the antioxidant NAC could successfully rescue HEI-OC-1 cells from TRMU downregulation-induced cell death and apoptosis (Fig. 6), and this suggested that ROS accumulation contributed to the increased sensitivity to neomycin injury in HEI-OC-1 cells after TRMU downregulation.

Mitochondria play a crucial role in cell metabolism, and apoptosis is closely linked to several events associated with mitochondrial dysfunction, including decreased mitochondrial membrane potential, increased ROS level, mitochondrial translation defects, and an increase in mtDNA copy number^44^,^45^. In sensory hair cells, the high oxygen consumption of mitochondria makes them sensitive to noise and ototoxic drug injury^26^, and aminoglycoside-induced hair cell loss is commonly linked with mitochondrial dysfunction and the accumulation of ROS^5^,^46^. Previous studies have shown that the accumulation of ROS triggers mitochondrial depolarization, initiates apoptosis, and leads to increased mtDNA copy number^37^,^47^. ROS accumulation also triggers changes in mitochondrial membrane permeability, which causes the loss of mitochondrial membrane potential^48^. The reduction of the mitochondrial transmembrane potential is one of the main features of cell apoptosis. The reduction of the mitochondrial membrane potential results in mitochondrial permeability transition pore opening that allows for the release of apoptotic factors from the mitochondria into the cytosol.

The mouse TRMU protein localizes in the mitochondria and is ubiquitously expressed, especially in tissues that require high oxidative metabolism^29^, and it has been suggested that *TRMU* deletion decreases the stability of mitochondrial tRNA and that this accounts for the translational deficiencies in mtDNA-encoded proteins that are essential parts of the respiratory chain^18^. However, the role of TRMU in regulating mitochondrial function after neomycin injury in HEI-OC-1 cells remains uninvestigated. In this study, we took advantage of TEM, immunohistochemistry, and flow cytometry to demonstrate that downregulation of TRMU dramatically increases the mitochondrial structural abnormalities and decreases the mitochondrial membrane potential of HEI-OC-1 cells after neomycin injury (Fig. 4). We also showed that treatment with the antioxidant NAC could rescue the TRMU downregulation-induced mitochondrial dysfunction of HEI-OC-1 cells (Fig. 7), suggesting that ROS accumulation is also involved in the increased mitochondrial dysfunction in HEI-OC-1 cells after TRMU downregulation. However, downregulation of TRMU failed to change the mtDNA copy number after neomycin injury (Fig. 5), suggesting that TRMU regulates mitochondrial function independently of mtDNA copy number after neomycin injury.

ROS accumulation causes hair cell damage upon treatment with ototoxic drugs such as aminoglycosides. Aminoglycosides lead to increased ROS, and antioxidant genes are subsequently upregulated to counteract the accumulation of ROS; thus the balance between prooxidant and antioxidant genes is critical for the accumulation rate of ROS. Many genes are coordinated to regulate the balance between ROS scavenging and production. In this study, we found that downregulation of TRMU significantly decreased the expression of three crucial antioxidant genes (*SOD*, *GSR*, and *GLRX*^49^) in HEI-OC-1 cells after neomycin injury (Fig. 5). This indicates that the inhibition of antioxidant genes causes changes in the antioxidant-prooxidant balance that leads to further accumulation of ROS. NAC, which is an important precursor to many antioxidants, plays a key role in attenuating oxidative stress by decomposing the peroxides and replenishing intracellular glutathione stores^50^. In this study, treatment with the ROS scavenger NAC rescued the increased mitochondrial dysfunction and apoptosis in HEI-OC-1 cells caused by TRMU downregulation after neomycin injury (Figs 6 and 7). Together, these results suggest that TRMU downregulation reduces the expression of antioxidant genes and leads to elevated ROS levels, which further contribute to mitochondrial dysfunction and apoptosis in HEI-OC-1 cells after neomycin injury.

In summary, we provide the first report that TRMU is expressed in mouse cochlear hair cells and in hair-cell-like HEI-OC-1 cells, and we demonstrate that TRMU plays an important role in regulating the sensitivity of HEI-OC-1 cells to neomycin-induced damage by preventing apoptosis and mitochondrial dysfunction of HEI-OC-1 cells after neomycin injury. We also demonstrate that the reduced expression of antioxidant genes after TRMU downregulation leads to increased ROS levels, which further contribute to the apoptosis and mitochondrial dysfunction induced by TRMU downregulation. Our findings provide new insights for novel therapeutic strategies for preventing hair cell death after drug-induced ototoxicity.

## Materials and Methods

### Cell culture

HEI-OC-1 cells were grown in DMEM supplemented with 10% FBS and 100 IU/ml penicillin (Sigma-Aldrich, St. Louis, USA)^27^. The cells were grown at 33 °C with 10% CO_2_ and subcultured at 80% confluence using 0.25% trypsin/EDTA (Life Technologies, Waltham, USA). Neomycin (Sigma-Aldrich) was used at a final concentration of 10 mM to damage the HEI-OC-1 cells, and N-acetyl-L-cysteine (NAC, Sigma-Aldrich) was used to inhibit the ROS accumulation.

### Real-time PCR

Total RNA was extracted from HEI-OC-1 cells with ExTrizol Reagent (PR910, Protein Biotechnology, Beijing, China) and reverse transcribed to cDNA by using cDNA Synthesis Kits (Thermo Fisher Scientific, Waltham, USA) according to the manufacturer’s protocol. Three marker genes (*ND1*, *COI*, and *COII*) – which represent mtDNA copy number – were measured by qPCR.

We performed qPCR on an Applied Biosystems CFX96 real-time PCR System (Bio-Rad, Hercules, USA) using the FastStart Universal SYBR Green (Rox) qPCR Master Mix (Roche, Basel, Switzerland). Validated primers were designed for each targeted mRNA or DNA (Supplementary Table S1). qPCR conditions were an initial denaturing step of 15 s at 95 °C followed by 40 cycles of 15 s denaturation at 95 °C, 60 s annealing at 60 °C, and 20 s extension at 72 °C. The mRNA expression values were normalized to the mRNA expression of *ACTB* and *GAPDH*. The results were calculated using the comparative cycle threshold (ΔΔCt) method.

### siRNA transfection in HEI-OC-1 cells

Three TRMU-specific rat siRNAs (GenePharma, Shanghai, China) were designed to knock down the expression of TRMU in HEI-OC-1 cells. An siRNA encoding a nonsense sequence was designed as the negative control. HEI-OC-1 cells were cultivated to 50% confluence in culture medium then washed with phosphate buffered saline (PBS). The medium was replaced 6 h later with Opti-MEM (Thermo Fisher Scientific) for transfection. HEI-OC-1 cells were transfected with TRMU-siRNA or control-siRNA according to the manufacturer’s instructions. The Opti-MEM medium was replaced 6 h later with DMEM containing fetal bovine serum. Cells were cultivated with neomycin after transfection for 36 h and then collected for western blot, CCK-8, immunofluorescence, and flow cytometry assays. The following siRNA was used to knock down the expression of TRMU: siRNA-TRMU-206, sense 5′-GCAUUUGUGCUGCUGACAATT-3′ and antisense 5′-UUGUCAGCAGCACAAAUGCTT-3′; siRNA-TRMU-402, sense 5′-CCAUUAUGCUGUGGACAAUTT-3′ and antisense 5′-AUUGUCCACAGCAUAAUGGTT-3′; siRNA-TRMU-575, sense 5′-GCUUUAAAGACCAGACCUUTT-3′ and antisense 5′-AAGGUCUGGUCUUUAAAGCTT-3′; negative control sense 5′-UUCUCCGAACGUGUCACGUTT-3′; and antisense 5′-ACGUGACACGUUCGGAGAATT-3′. The negative control siRNA and siRNA-TRMU were mixed with Lipofectamine 2000 (Invitrogen, Waltham, USA) at a final concentration of 500 nmol/L siRNA in medium.

### Immunofluorescence

The immunohistochemistry was performed using the Fast ImmunoCytoChemistry Staining Kit (BPICC30-1KT, Protein Biotechnologies, USA). Tetramethylrhodamine ethyl ester perchlorate (TMRE, Sigma-Aldrich), anti-Caspase 3 antibody (Cell Signaling Technology Inc., Danvers, USA), Mito-SOX Red (Life Technologies, Waltham, USA), anti-TRMU antibody (Santa Cruz Biotechnology, Dallas, USA), anti-Myosin7a antibody (Proteus Bioscience, San Diego, USA), phalloidin (Sigma-Aldrich), and DAPI were used to analyze mitochondrial membrane potential, detect apoptotic cells, measure reactive oxygen species (ROS), and stain TRMU protein, hair cells, microfilament structures, and nuclei, respectively. HEI-OC-1 cells were grown in four-well culture dishes filled with DMEM culture medium.

For TMRE and Mito-SOX staining, the culture medium was removed from the dish and the samples were washed with PBS. HEI-OC-1 cells were incubated in DMEM culture medium containing TMRE or Mito-SOX for 30 min. After staining, the cells were washed in prewarmed PBS and imaged with a confocal microscope (LSM700; Zeiss, Heidenheim, Germany). A TUNEL Kit (Roche, Indianapolis, USA) was used to detect apoptotic cells according to the manufacturer’s instructions.

For other antibody staining, primary antibodies were added and incubated for 10 h (4 °C) at a dilution of 1:400–1:1000 after HEI-OC-1 cells were cultured, fixed with 4% polyoxymethylene for 1 h, and permeabilized with 0.5% Triton X- 100 for 1 h. The cells were washed three times with PBST and incubated for 1 h (37 °C) with red or green fluorescence-conjugated immunoglobulin G secondary antibody (Abcam, Cambridge, UK), phalloidin, or DAPI. The cells were imaged with a confocal microscope.

### Cell number analysis

Cells were trypsinized, collected by centrifugation at 800 rpm for 5 min, resuspended in culture medium, and plated on 96-well plates at 2,000 cells/well with three replicates. After 24 h incubation, neomycin was added at different concentrations (controls received a similar volume of DMEM). Cell numbers were counted with the CCK-8 Cell Counting Kit (CC201, Protein Biotechnology, Beijing, China) at different time points after incubation.

### Electron microscopy

Cells were collected by centrifugation after trypsinization and immediately fixed in 2.5% glutaraldehyde (Sigma-Aldrich) for 24 h and then in 1% osmic acid (Sigma-Aldrich) for 1–2 h, dehydrated with acetone (Sinopharm Chemical Reagent, Shanghai, China), and embedded in araldite CY 212 (TAAB, Aldermaston, UK). The ultrathin sections were stained with alcoholic uranyl acetate (Polysciences, Warrington, USA) and alkaline lead citrate (Sigma-Aldrich), washed gently with distilled water, and observed with a JEM 1230 transmission electron microscope (JEOL Ltd, Tokyo, Japan).

### Flow cytometry

TMRE and Mito-SOX were used to determine the mitochondrial membrane potential and to analyze ROS production. HEI-OC-1 cells were trypsinized, collected by centrifugation at 800 rpm for 5 min, and resuspended in solution containing TMRE or Mito-SOX for 10 min followed by washing with PBS and analysis by flow cytometry (FACSCanto, BD, San Jose, USA). If required, the cells were pretreated with NAC to lower the ROS level.

An Annexin V kit (BD) was used for apoptosis analysis. The HEI-OC-1 cells were trypsinized, collected by centrifugation at 800 rpm for 5 min, and then washed twice with PBS and resuspended in 1× binding buffer at a concentration of 1 × 10^6^ cells/ml. Annexin V-FITC and PI were added and gently mixed with the cells and incubated for 10–30 min at room temperature in the dark. The cells were analyzed by flow cytometry as soon as possible, and all experiments were repeated at least three times.

### Western blot

The HEI-OC-1 cells were lysed with RIPA Lysis Buffer (PP109, Protein Biotechnology). Protein concentrations were measured using a BCA Protein Quantification Kit (PP202, Protein Biotechnology) according to the manufacturer’s instructions using GAPDH as the reference protein. TRMU was monitored using anti-TRMU rabbit polyclonal antibody (Santa Cruz Biotechnology), and GAPDH was measured using a mouse monoclonal antibody (Abcam). Peroxidase-conjugated goat anti-rabbit (or anti-mouse) immunoglobulin G (Abcam) was employed as the secondary antibody. The proteins were bound to polyvinylidene fluoride membranes and detected using a SuperSignal West Dura chemiluminescent substrate kit (Thermo Scientific) according to the manufacturer’s instructions. Semi-quantification of the western blot results was performed using Image J to measure the intensities of the bands.

### Statistical analysis

All the data are shown as the mean ±S.D. Statistical analyses were conducted using Microsoft Excel and GraphPad Prism6 software. For all experiments, *n* represents the number of replicates. Two-tailed, unpaired Student’s *t*-tests were used to determine statistical significance when comparing two groups, and one-way ANOVA followed by a Dunnett’s multiple comparisons test was used when comparing more than two groups. A value of *p* < 0.05 was considered to be statistically significant.

## Additional Information

**How to cite this article**: He, Z. *et al*. Reduced TRMU expression increases the sensitivity of hair-cell-like HEI-OC-1 cells to neomycin damage in vitro. *Sci. Rep.* **6**, 29621; doi: 10.1038/srep29621 (2016).

## Supplementary Material

Supplementary Information
