# Supplementary material for: Reduced TRMU expression increases the sensitivity of hair-cell-like HEI-OC-1 cells to neomycin damage in vitro
Source: Sci Rep. 2016 Jul 13;6:29621. doi: 10.1038/srep29621 (PMC4942793; doi:10.1038/srep29621)
Supplement: Supplementary Information [file srep29621-s1.docx]

**Reduced TRMU expression increases the sensitivity of hair-cell-like HEI-OC-1 cells to neomycin damage in vitro**

Abbreviated title: TRMU regulates HEI-OC-1 cell survival after neomycin exposure

Zuhong He ^a,1^, Shan Sun^b,1^, Muhammad Waqas^a,c,d^, Xiaoli Zhang^f^, Fuping Qian^a,c,d^, Cheng Cheng ^a,c,d^, Mingshu Zhang^g^, Shasha Zhang^a,c,d^, Yongming Wang^e^, Mingliang Tang^a,c,d^, Huawei Li^b,e^*, Renjie Chai^a,c,d^*

^a^State Key Laboratory of Bioelectronics, Institute of Life Sciences, Southeast University, Nanjing 210096, China

^b^Department of Otorhinolaryngology, Hearing Research Institute, Affiliated Eye and ENT Hospital of Fudan University, Shanghai 200031, China

^c^MOE Key Laboratory of Developmental Genes and Human Disease, Institute of Life Sciences, Southeast University, Nanjing 210096, China

^d^Co-Innovation Center of Neuroregeneration, Nantong University, Nantong 226001, China

^e^Institutes of Life Sciences, Fudan University, Shanghai 200032, China

^f^Department of Otolaryngology, Affiliated Drum Tower Hospital of Nanjing University Medical School, Nanjing 210008, China

^g^Medical School, Southeast University, Nanjing 210096, China

^1^ These authors contributed equally to this work.

***Corresponding authors:**

Renjie Chai, Ph.D., Co-Innovation Center of Neuroregeneration, Key Laboratory for Developmental Genes and Human Disease, Ministry of Education, Institute of Life Sciences, Southeast University, Nanjing 210096, China, Tel/Fax: 86-25-83790971, renjiec[@seu.edu.cn](mailto:lihuawei63@gmail.com)

And

Huawei Li, M.D., Department of Otorhinolaryngology, Affiliated Eye and ENT Hospital of Fudan University, Room 611, Building 9, No. 83, Fenyang Road, Xuhui District, Shanghai, China, 200031, Tel: 86-21-64377134-669, Fax: 86-21-64377151, [lihuawei63@gmail.com](mailto:lihuawei63@gmail.com)

**
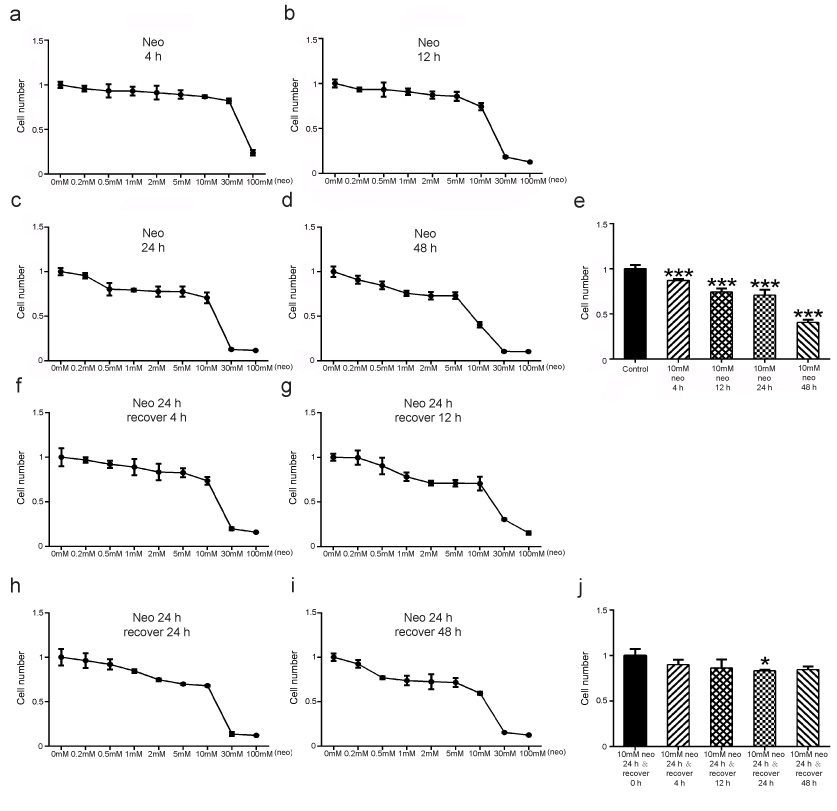
**

**Supplemental figure 1.** Analysis of the [sensitivity](http://www.baidu.com/link?url=QY9woB0Yyj4smO9lW5tsP51FNRsfegAbCfSebJOgsQlphGVFGWX5bHz5aVoJ0Zdgfx_neqmtQW5lyeSIFmKS7G8yXYlFkskKkt8u12jL8HuCgrlOeYlPEUmYVkV7Yugj) of HEI-OC-1 cells to neomycin. (a–d) After treatment with different concentrations of neomycin (from 0.2 mM to 100 mM) for different periods of time (from 4 h to 48 h), the number of live cells was measured with the CCK-8 kit. (e) Treatment with 10 mM neomycin significantly reduced the number of live cells at different treatment times. (f–i) After 24 h of treatment with different concentrations of neomycin (from 0.2 mM to 100 mM), the HEI-OC-1 cells were allowed to recover for 4 h to 48 h. (j) The number of live cells decreased when the recovery time increased after treatment with 10 mM neomycin for 24 h. For all experiments, the values for the normal controls were set to 1. **p* < 0.05, ***p* < 0.01, ****p* < 0.001.

**
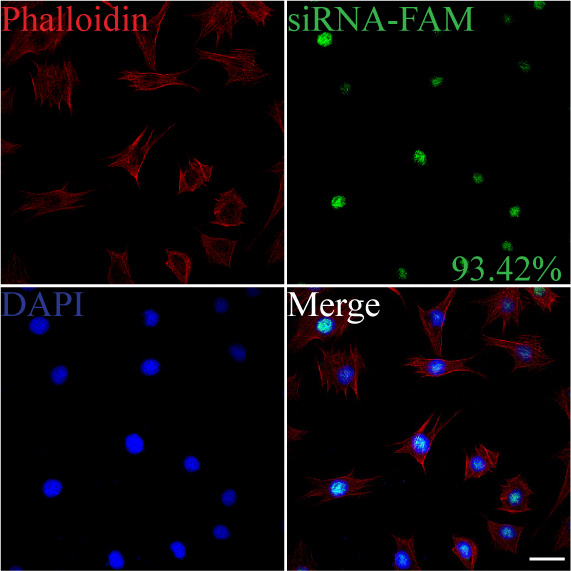
**

**Supplemental figure 2.** Measurement of the efficiency of the siRNA transfection system. The proportion of FAM/DAPI double-positive cells calculated after HEI-OC-1 cells were transfected with FAM-siRNA. Scale bars = 20 μm.


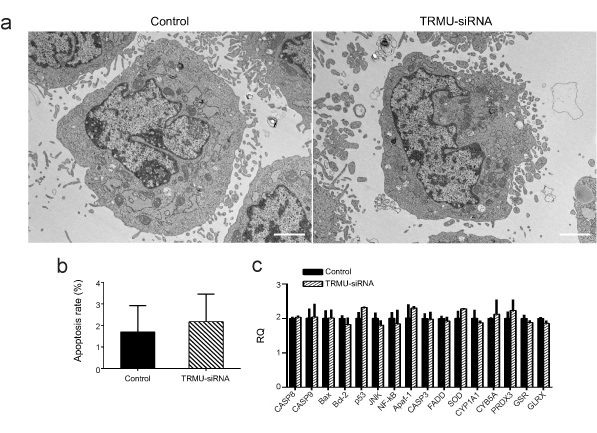


**Supplemental figure 3.** Analysis of the expression of mitochondrial function and apoptosis-related genes after downregulation of TRMU without neomycin treatment. (a) Transmission Electron Microscope analysis (TEM) showed that there are no mitochondrial structural changes in HEI-OC-1 cells after downregulation of TRMU compared with control cells. (b) Quantification of the data in a. (c) The mRNA levels of genes related to apoptosis and oxidation-reduction reactions were analyzed by qPCR. The results showed that no significant changes were observed in HEI-OC-1 cells after siRNA transfection without neomycin treatment. For qPCR experiments, the values for the normal controls were set to 1. Scale bars = 2 μm.


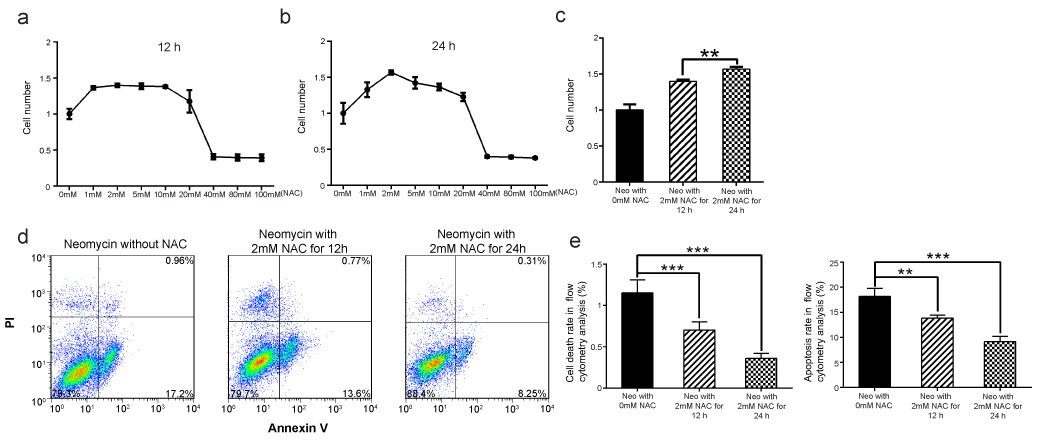


**Supplemental figure 4.** Analysis of the ability of NAC to block the increase in ROS levels in HEI-OC-1 cells treated with neomycin. (a and b) After pretreatment with different concentrations of NAC (from 1 mM to 100 mM) for different periods of time (12 h and 24 h), the number of live cells was measured with the CCK-8 kit. The results showed that the proportion of apoptotic cells was significantly reduced when pretreated with 2 mM NAC. (c) The number of live cells reached the highest point when treated with 2 mM NAC for 24 h. (d) Apoptosis analysis by flow cytometry. (e) The proportions of dead cells and early apoptotic cells after neomycin treatment were significantly reduced after transfection with NAC for 12 h or 24 h. For all experiments, the values for the normal controls were set to 1. **p* < 0.05, ***p* < 0.01, ****p* < 0.001.

**
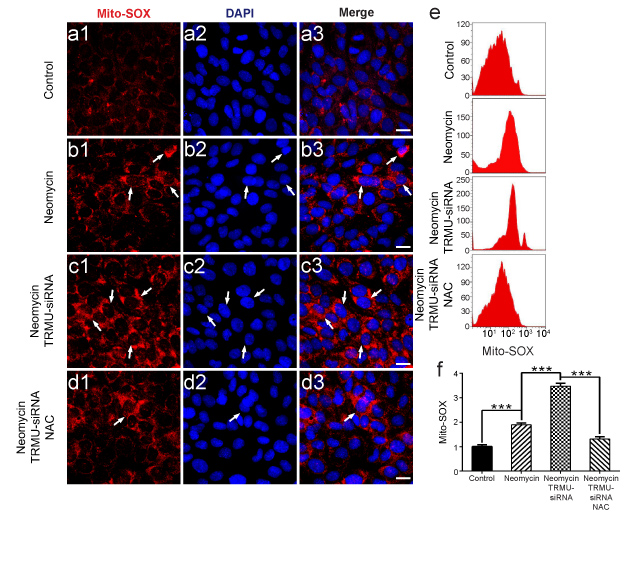
**

**Supplemental figure 5.** The influence of antioxidants on the ROS levels of TRMU-downregulated HEI-OC-1 cells after neomycin treatment. (a–d) Four different groups of HEI-OC-1 cells were labeled with the Mito-SOX staining kit. The results showed that the ROS levels were significantly decreased after pretreatment with NAC; however, the expression was slightly increased compared with the control cells. (e) Flow cytometry data confirmed the results in a–d. (f) Quantification of the flow cytometry data. For all experiments, the values for the normal controls were set to 1. Scale bars = 20 μm, **p* < 0.05, ***p* < 0.01, ****p* < 0.001.
